# From Microbes to Memories: Challenges and Future Perspectives Regarding the Gut-Brain Axis for Improved Cognitive Health in Alzheimer’s

**DOI:** 10.7759/cureus.52795

**Published:** 2024-01-23

**Authors:** Carlos D Franco, Raja Subhash Sagar, Syed Faqeer Hussain Bokhari

**Affiliations:** 1 Medicine, Universidad Laica Eloy Alfaro de Manabí, Manta, ECU; 2 Medicine, Liaquat University of Medical and Health Sciences, Jamshoro, PAK; 3 Surgery, King Edward Medical University, Lahore, PAK

**Keywords:** metabolites, neuroinflammation, microbiota dysbiosis, cognitive health, alzheimer's disease, gut-brain axis

## Abstract

The gut-brain axis, a bidirectional communication network between the gastrointestinal tract and the central nervous system, regulates various physiological processes crucial for health, including immune response, metabolism, and neurotransmitter production. In the context of neurodegenerative diseases, especially Alzheimer's disease (AD), understanding the intricate connection of the gut-brain axis has gained significance. Disturbances along this axis have been implicated in neurodegenerative diseases, emphasizing its role in AD pathogenesis. Microbiota dysbiosis, influenced by diet, lifestyle, and genetics, contributes to altered gut permeability, leading to protein dyshomeostasis, astroglial activation, neuroinflammation, and cognitive decline. Understanding these mechanisms is crucial for developing interventions to restore a healthy gut microbiota and potentially mitigate AD-related cognitive decline.

The bidirectional communication along the gut-brain axis involves microbial metabolites, influencing oxidative stress, protein aggregation, and other pathways linked to neuroprotection. Modulating the gut microbiota through dietary changes, prebiotics, probiotics, or fecal microbiota transplantation emerges as a promising approach to target cognitive decline in AD. Despite progress, challenges persist, including the correlational nature of studies, the complexity of the gut microbiome, and variations in methodologies. Standardization is essential for reliable findings and the identification of biomarkers associated with AD. Unanswered questions warrant further exploration, particularly in understanding specific mechanisms, the temporal dynamics of microbiota changes, and the influence of diet and lifestyle on the gut-brain axis in AD. Future perspectives involve promising therapeutic interventions targeting the gut-brain axis, emphasizing personalized medicine to optimize outcomes based on individual variations in the gut-brain axis characteristics.

## Editorial

Introduction

The gut-brain axis represents a bidirectional communication network between the gastrointestinal tract and the central nervous system. This intricate connection plays a pivotal role in regulating various physiological processes, including immune response, metabolism, astroglial homeostasis, and neurotransmitter production. Understanding its significance has become increasingly relevant, particularly in the context of neurodegenerative diseases like Alzheimer's disease (AD). The gut-brain axis involves complex signaling pathways between the gut and the brain, influencing cognitive and emotional functions. This bidirectional communication occurs through neural, endocrine, and immune pathways, facilitated by the gut microbiota. The gut microbiota, comprising trillions of microorganisms, plays a crucial role in maintaining homeostasis and influencing various aspects of health, including brain function [1].

AD, the most prevalent form of dementia, primarily affects regions of the brain responsible for memory, thought, and language [2]. It manifests as a progressive decline in cognitive function, impacting daily life. The epidemiology of AD is marked by a current diagnosis of approximately 50 million people with dementia, a number projected to escalate to 131 million by 2050, posing significant societal implications and a global social cost of US$1.3 trillion [1]. Despite extensive research, the exact etiology of AD remains elusive, emphasizing the need for exploring unconventional avenues, such as the gut-brain axis, to comprehend the disease's complexity. Recent studies highlight disturbances along the brain-gut-microbiota axis as potential contributors to neurodegenerative diseases, including AD [2]. The gut microbiota's influence on neuroinflammation, neuronal function, and amyloid-beta deposition underscores its significance in cognitive health. Exploring this axis provides a novel perspective on AD pathogenesis and potential therapeutic interventions.

This editorial aims to synthesize existing knowledge on the gut-brain axis and its relevance to cognitive health in AD. By examining the latest research, we seek to shed light on the intricate interplay between gut microbiota, neuroinflammation, and AD pathology. Through this exploration, we aspire to contribute to a deeper understanding of potential therapeutic targets for mitigating cognitive decline in AD.

Microbiota dysbiosis in AD

The bidirectional relationship between the gut microbiota and the brain, known as the brain-gut-microbiota axis, plays a crucial role. Disruptions in this axis may lead to altered gut permeability, facilitating the translocation of proinflammatory molecules and microbial products into the systemic circulation, and contributing to neuroinflammation. Several factors contribute to microbiota dysbiosis in the context of AD. These include diet, lifestyle, and genetic factors [3]. Moreover, the genetic correlation between AD and gut dysbiosis suggests a potential genetic predisposition to alterations in the gut microbiota [3]. Understanding these mechanisms is crucial for developing targeted interventions aimed at restoring a healthy gut microbiota and potentially mitigating AD-related cognitive decline.

Bacterial amyloids, notably curli produced by* Escherichia coli *(*E. coli*), contribute significantly to gut microbiota composition [2]. Recent studies show that exposure to bacterial amyloid proteins may prime the immune system, leading to enhanced immune responses in both the gut and brain, influencing neurodegenerative processes such as alpha-synuclein deposition. Additionally, lipopolysaccharides (LPS) from bacteria, particularly *E. coli,* play a role in neuroinflammation and amyloid fibrillogenesis, with LPS presence detected in brain lysates from AD patients, supporting the concept of molecular mimicry [2]. Furthermore, gut inflammation, indicated by elevated fecal calprotectin, and gut barrier dysfunction are associated with neurodegeneration, linking intestinal processes to AD pathophysiology [2,4]. The gut microbiota's role in modulating neuroinflammatory responses and influencing the accumulation of pathological proteins, such as molecular mimicry of beta-amyloid, highlights its impact on AD progression. Understanding these intricate relationships is crucial for developing therapeutic strategies targeting the gut microbiota to potentially slow down or prevent neurodegenerative processes in AD.

Gut-brain axis and cognitive health

Numerous studies have provided compelling evidence linking alterations in gut microbiota composition to cognitive decline in AD [1,2]. These alterations in microbial composition may contribute to the neuroinflammatory processes and the accumulation of pathological proteins associated with cognitive impairment in AD. Findings suggest that gut microbial metabolites play a crucial role in influencing brain function. Metabolites produced by the gut microbiota can impact oxidative stress, aggregation of neurotoxic proteins, and other pathways linked to neuroprotection. The bidirectional communication along the gut-brain axis involves these metabolites, highlighting their potential influence on cognitive health. Understanding the gut-brain axis's role in cognitive health opens avenues for potential therapeutic interventions. Modulating the gut microbiota through dietary changes, prebiotics, probiotics, or fecal microbiota transplantation emerges as a promising approach [1,5]. Targeting the gut-brain axis holds promise in mitigating cognitive decline and improving the overall quality of life for individuals affected by neurodegenerative disorders.

Current challenges and unanswered questions

As the association between the gut-brain axis and AD is gaining attention, several limitations in existing research must be acknowledged. Many studies, though insightful, often present a correlational nature, making it challenging to establish causation [1-5]. The gut microbiome's complexity and dynamic nature pose challenges in deciphering specific microbial contributions to AD pathology. Additionally, variations in study designs, sample sizes, and participant demographics further complicate the interpretation of results.

Standardization of methodologies is imperative for advancing our understanding of the gut-brain axis in AD. Research trends indicate a need for consistent approaches in sample collection, processing, and data analysis to ensure reproducibility and comparability across studies [4]. Standardized protocols will enhance the reliability of findings and facilitate the identification of robust biomarkers or microbial signatures associated with AD. Establishing a common framework will also aid in integrating data from different studies, fostering collaborative efforts in unraveling the intricate relationship between the gut and the brain in the context of AD.

Despite significant progress, numerous questions remain unanswered, necessitating further exploration. The specific mechanisms through which gut microbiota influence neuroinflammation, amyloid beta aggregation, and oxidative stress in AD need elucidation [1]. Understanding the temporal dynamics of microbiota changes and their impact on the different stages of AD progression is crucial for targeted interventions. Moreover, the role of individual microbial species or groups in modulating AD pathology requires a deeper investigation. Identifying key microbial metabolites and their precise mechanisms of action on brain function will contribute to developing targeted therapeutic strategies. The influence of diet and lifestyle on the gut-brain axis in AD remains an understudied aspect, warranting comprehensive investigations to delineate the impact of external factors on microbial composition and subsequent neurological outcomes.

Future perspectives

Recent research, as highlighted in various studies [1,2,5], has shown promising avenues for therapeutic interventions targeting the gut-brain axis in AD. Probiotic treatments have demonstrated the ability to modulate gut microbiota, offering a potential avenue for influencing AD pathology. Understanding the mechanisms by which specific microbial strains impact neuroinflammation and amyloid beta aggregation opens doors for the development of targeted probiotic therapies. Future exploration may focus on clinical trials to validate the efficacy of these interventions and to identify optimal treatment regimens.

The integration of gut health considerations into AD treatment strategies represents a paradigm shift in approaching neurodegenerative diseases. Considering the bidirectional communication between the gut and the brain, lifestyle modifications and dietary interventions aimed at promoting a healthy gut microbiome could become integral components of AD management. Healthcare practitioners may incorporate personalized nutrition plans, emphasizing prebiotics and probiotics, to support gut health and potentially alleviate AD symptoms. Collaborative efforts between neurologists, gastroenterologists, and nutritionists are crucial to developing comprehensive treatment strategies that encompass both the neurological and gastrointestinal aspects of this disease.

The gut-brain axis exhibits considerable individual variations, necessitating a personalized medicine approach in AD management. Understanding how specific microbial compositions contribute to AD progression allows for tailored interventions based on an individual's microbiome profile. This approach considers factors such as age, genetics, and lifestyle, providing a more nuanced understanding of the disease. Precision medicine strategies may involve microbiome analysis to guide personalized dietary recommendations, probiotic prescriptions, or other targeted interventions. Such tailored approaches acknowledge the heterogeneity of AD and strive to optimize therapeutic outcomes based on individual gut-brain axis characteristics.
